# Medical Marijuana Documentation Practices in Patient Electronic Health Records: Retrospective Observational Study Using Smart Data Elements and a Review of Medical Records

**DOI:** 10.2196/65957

**Published:** 2024-12-23

**Authors:** Donielle Beiler, Aanya Chopra, Christina M Gregor, Lorraine D Tusing, Apoorva M Pradhan, Katrina M Romagnoli, Chadd K Kraus, Brian J Piper, Eric A Wright, Vanessa Troiani

**Affiliations:** 1 Autism and Developmental Medicine Institute, Geisinger, Lewisburg, PA, United States; 2 Center for Pharmacy Innovation and Outcomes, Geisinger, Danville, PA, United States; 3 Department of Population Health Sciences, Geisinger, Danville, PA, United States; 4 Department of Emergency and Hospital Medicine, Lehigh Valley Health Network, Hazelton, PA, United States; 5 Department of Medical Education, Geisinger Commonwealth School of Medicine, Scranton, PA, United States; 6 Department of Bioethics and Decision Sciences, Geisinger, Danville, PA, United States

**Keywords:** cannabis, learning health system, Epic, prescription drug monitoring program, medical marijuana, electronic health records, physician, cannabis use, drug use, data sharing, patient care, legalization, dosage, chart review protocol, human data extraction, data collection

## Abstract

**Background:**

Medical marijuana (MMJ) is available in Pennsylvania, and participation in the state-regulated program requires patient registration and receiving certification by an approved physician. Currently, no integration of MMJ certification data with health records exists in Pennsylvania that would allow clinicians to rapidly identify patients using MMJ, as exists with other scheduled drugs. This absence of a formal data sharing structure necessitates tools aiding in consistent documentation practices to enable comprehensive patient care. Customized smart data elements (SDEs) were made available to clinicians at an integrated health system, Geisinger, following MMJ legalization in Pennsylvania.

**Objective:**

The purpose of this project was to examine and contextualize the use of MMJ SDEs in the Geisinger population. We accomplished this goal by developing a systematic protocol for review of medical records and creating a tool that resulted in consistent human data extraction.

**Methods:**

We developed a protocol for reviewing medical records for extracting MMJ-related information. The protocol was developed between August and December of 2022 and focused on a patient group that received one of several MMJ SDEs between January 25, 2019, and May 26, 2022. Characteristics were first identified on a pilot sample (n=5), which were then iteratively reviewed to optimize for consistency. Following the pilot, 2 reviewers were assigned 200 randomly selected patients’ medical records, with a third reviewer examining a subsample (n=30) to determine reliability. We then summarized the clinician- and patient-level features from 156 medical records with a table-format SDE that best captured MMJ information.

**Results:**

We found the review protocol for medical records was feasible for those with minimal medical background to complete, with high interrater reliability (κ=0.966; *P<*.001; odds ratio 0.97, 95% CI 0.954-0.978). MMJ certification was largely documented by nurses and medical assistants (n=138, 88.5%) and typically within primary care settings (n=107, 68.6%). The SDE has 6 preset field prompts with heterogeneous documentation completion rates, including certifying conditions (n=146, 93.6%), product (n=145, 92.9%), authorized dispensary (n=137, 87.8%), active ingredient (n=130, 83.3%), certifying provider (n=96, 61.5%), and dosage (n=48, 30.8%). We found preset fields were overall well-recorded (mean 76.6%, SD 23.7% across all fields). Primary diagnostic codes recorded at documentation encounters varied, with the most frequent being routine examinations and testing (n=34, 21.8%), musculoskeletal or nervous conditions, and signs and symptoms not classified elsewhere (n=21, 13.5%).

**Conclusions:**

This method of reviewing medical records yields high-quality data extraction that can serve as a model for other health record inquiries. Our evaluation showed relatively high completeness of SDE fields, primarily by clinical staff responsible for rooming patients, with an overview of conditions under which MMJ is documented. Improving the adoption and fidelity of SDE data collection may present a valuable data source for future research on patient MMJ use, treatment efficacy, and outcomes.

## Introduction

Since 1996, states across the United States have legalized cannabis for medical use [1]. The legalization of cannabis or medical marijuana (MMJ) adds another layer of complexity to comprehensive patient care, as treatment with MMJ is managed outside of traditional health care management and documentation systems [2]. In Pennsylvania, MMJ is not currently “prescribed”—rather, a physician with appropriate privileges must certify that a given patient has 1 of 24 serious medical conditions [3,4]. This certification can then be used to register and obtain an MMJ card for use at dispensaries, where a health care provider (typically a pharmacist) is available for consultation or advisement and a patient care specialist assists with product selection for a given symptom or diagnosis [5].

Although MMJ use in Pennsylvania requires certification by a qualified physician, there is not a standardized integration of data from MMJ purchases with health systems, such as those that exist for prescription drugs [6]. While most states with an MMJ program use a documentation system for the dispensing of MMJ to patients, these systems are not uniformly integrated with state-managed prescription drug monitoring programs, which are often linked to health system records. The integration of prescription drug monitoring programs has been shown to improve health outcomes by allowing for comprehensive and coordinated patient care [7], with more direct integration of the databases associated with increased use by clinicians [8].

Electronic health record (EHR) systems digitize patient health records to allow for information sharing between providers, institutions, and insurance companies to improve care continuity and track billing based on the standards for meaningful use outlined in the Health Information Technology for Economic and Clinical Health Act of 2009 [9,10]. The records contain diagnoses, encounter documentation, provider notes, scans and laboratory results, medications, and patient communication as well as demographic details. Most EHRs contain a standard set of input options that have shown utility for patient care and billing purposes, typically focused on capturing details associated with a specific visit (eg, physical examination and current medication review). Other components of a patient’s record are important for longitudinal care—including a social history section where a range of lifestyle and behavior choices can be documented, as well as the familial history of disease [11]. Documentation systems can be helpful to care teams because they prompt patient conversations surrounding these topics and encourage consistent documentation to facilitate longitudinal care [12,13].

In addition to standardized data entry and workflows available within EHRs, many customization options for data entry exist [14,15]. One EHR system, Epic, allows for smart data elements (SDEs) to be triggered by built-in text or phrases that load preset text with components that can be edited by health care professionals [16,17]. This improves data entry efficiency and creates coded variables, from which data can be extracted in an automated way [18]. Each SDE element can have preset options or contain free text cells, in which providers can list multiple responses. The SDE (triggered by Epic SmartText or Epic SmartPhrase or located in flowsheet rows) can be linked to other EHR items like a problem list diagnosis or encounter note. For MMJ use documentation, a customized SDE was created, with individual components mimicking those on product labels from MMJ dispensaries [5]. After entering the SmartText, the Epic user will see each of the individual components (in this case—certifying provider, authorized dispensary, certifying condition, dosage product provided, dose, and active ingredient) and can input text into each section. Even with structured documentation variables in place, implementation and use can be inconsistent [19].

EHR systems also serve as a wealth of information for retrospective research [20,21]. Large databases can be generated from the fields created and entered by clinical providers and hospital staff. Variables within discrete fields are typically easier to extract from the EHR in an automated way, but there is a great deal of potentially useful information within free text fields that require more sophisticated methods of data extraction. One method to extract useful clinical information from EHR notes is to use a review protocol for medical records, in which a human reviewer reads the medical record and documents relevant information. We have previously developed review protocols for medical records that enabled the consistent extraction of relevant text that informed opioid use disorder severity [22] and autonomic arousal dimensions [23], but many different approaches for reviewing medical records exist, and best practice can be determined based on an individual use case or purpose for analysis [24-26]. To ensure high fidelity of the extracted data, it is necessary to develop protocols that enable a reviewer to identify specific responses that can be replicated by other reviewers [27,28].

In this exploratory study, we implemented a systematic protocol for reviewing medical records to document how the MMJ SDE is being used within Geisinger. We developed and performed this protocol on a random subset (n=200) of the total Geisinger patient population with an existing marijuana SDE (n=2133). The primary questions driving this research were (1) whether the MMJ SDE was consistently capturing MMJ-related information, (2) where this SDE was being used within the medical record, and (3) whether this SDE was serving its intended purpose of making the patient’s use of MMJ easily accessible and available to clinicians across our integrated care system.

## Methods

### Study Sample Participants

The work described here was approved by our institutional review board. Reviews of medical records were completed with a waiver of informed consent. Patients who had an Epic SDE coded for marijuana use (n=2133) between August 1, 2017, and June 29, 2022, and were 18 years and older of age were identified and eligible for reviewing medical records as part of a larger study aim. A random number generator was used to assign a value to each record and sorted numerically to obtain a sample cohort (n=5 for the initial development and n=200 [100 per reviewer] for full review of medical records). This number of 200 medical records was chosen based on common practices of completing at least 100 reviews of medical records in similar validation studies, along with feasibility considerations of study and personnel time constraints to perform the review. Following the review of medical records, it was realized that the 200 patients did not all have the same type of marijuana SDE. Rather, 156 patients in the cohort had the table-format MMJ SDE that listed 6 discrete elements (certifying provider name and location, certifying condition, dispensary, dose, product type, and active ingredient), while the remaining 44 patients had a second type of social history SDE that coded for marijuana use frequency, method of use, and last use date. As our primary goal was to assess whether the table-format SDE was a useful documentation tool for information that is part of patient registration for MMJ in Pennsylvania, we focused our analysis and summary on the 156 patients that contained the table-formatted MMJ SDE.

### Process for Reviewing Medical Records

We created a protocol for reviewing medical records with the goal of extracting information relevant to these primary research questions, with a secondary goal to optimize the protocol to ensure consistent data extraction across medical record reviewers. An iterative approach was used to create a list of variables that could easily be extracted from the EHR through manual review. An initial subset of 5 records was comprehensively reviewed by study team members to determine what MMJ-related data could be gleaned from the EHR and where in the EHR the desired information could be obtained. These records were reviewed by a clinical and research team to determine a consensus of desirable variables. A detailed workflow was created, highlighting specific areas of the patient record to search for the data of interest (problem list, encounters, scanned documents, laboratory orders, and search terms). Explicit wording and directions were created to guide the medical record reviewers through a systematic protocol. The final variables included 61 discrete fields with specific expected responses and 10 descriptive fields for notes and comments as needed. These variables included MMJ SDE documentation location and details, social history documentation, marijuana diagnoses, documenting provider and department information, primary and secondary diagnoses at the time of MMJ documentation, copies of the MMJ certification, relevant toxicological screen results, presence or absence of MMJ documentation on subsequent encounters, total EHR length, the first documentation of both MMJ interest and MMJ use, and any notable side effects (Textbox S1 in Multimedia Appendix 1).

**Figure 1.**
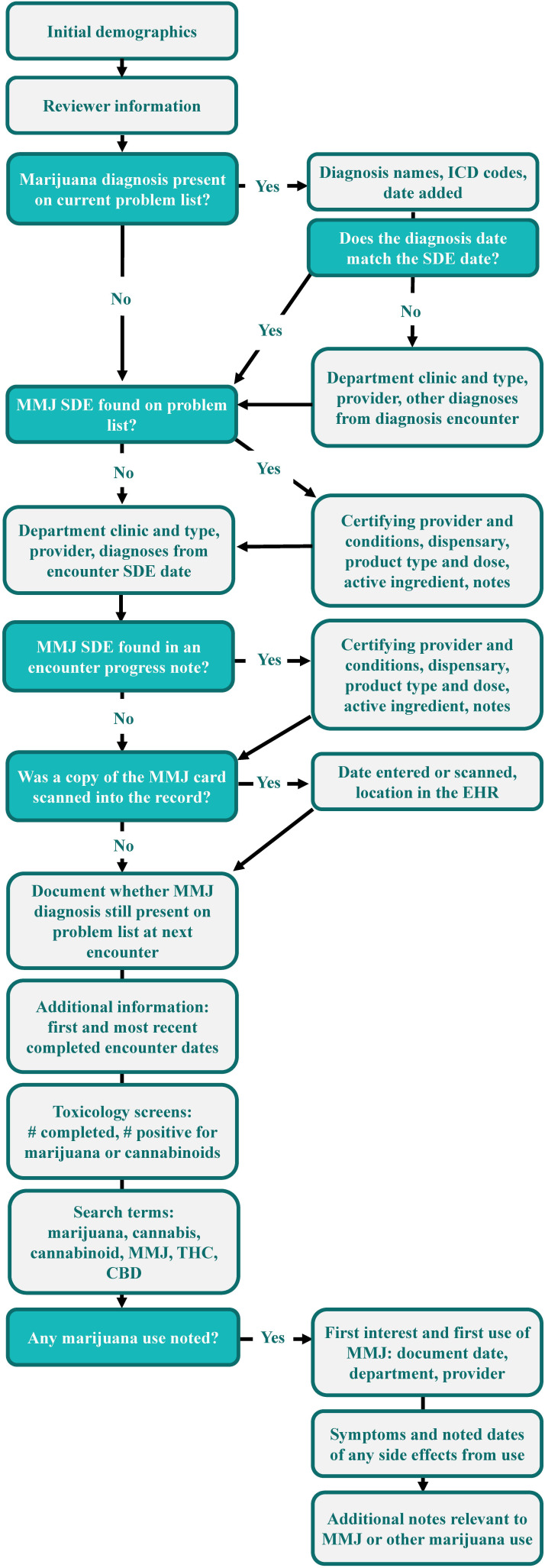
Systematic process by which reviewers identified responses and recorded data from patient records following detailed instructions on where to search for each specific field. Branching logic allowed sections to be added or skipped depending on the result of core questions to optimize review time and effort. CBD: cannabidiol; EHR: electronic health record; *ICD*: *International Classification of Diseases*; MMJ: medical marijuana; SDE: smart data element; THC: tetrahydrocannabinol.

A data capture tool was initially developed in Microsoft Excel to make it easily accessible for team members with limited data collection experience, as it does not require special permissions or training, and template changes can be implemented by someone without specific expertise. A detailed instruction manual with visual aids was also created to be a step-by-step walkthrough of the review process for medical records (eg, “Was a marijuana diagnosis present on the current problem list? Yes or No. If yes, please list the diagnosis name(s), *ICD* (International Classification of Diseases) codes and date added to the problem list in the subsequent columns. If no, please note “N/A” in the subsequent columns”; Figure 1). In total, 13 of the 61 discrete fields were identified as independent variables, under which branching logic added or skipped whole subsections of the review to streamline data collection. Once the instruction manual and data capture tools were created, the members of the team who would be completing the larger cohort for review of medical records were tasked with testing the process on that same pilot subset. The results were compared for similarity, and in instances of incongruity, edits were made to the instructions to clarify the expected outcome.

The finalized instruction manual was then used by 2 reviewers (AC and Julia Soares) to independently review all medical records (each patient’s medical record reviewed by 1 of the 2 primary reviewers). To determine interrater reliability, a third reviewer (DB) completed a duplicate review on a random subset of medical records (n=30), repeating the full review process while being blind to the previous reviewer’s documentation.

### Statistical Analyses

Interrater reliability was calculated on the 30 medical records reviewed by the third reviewer compared with that of the initial reviewer (comparison medical records). A standard interrater reliability calculation was applied by comparing the results of each of the 61 discrete variables between reviewers and dividing the number of congruent fields by the total number of discrete fields. As discrete responses could be affected by the independent variables, the same calculation process was applied to only those 13 independent variables. In both cases, range, mean, and SD were calculated.

As a measure of interrater reliability, Cohen κ calculations were also applied [29]. For each of the comparison medical records, the discrete fields of both the primary reviewer and the third reviewer were identified as 1 of 3 outcomes: the value matched the EHR following the instructions (F), the value did not match the EHR following the instructions (D), or the value was null (N). The outcomes of each paired response were then combined into a 2-letter label (FF, FD, FN, DF, DD, DN, NF, ND, and NN). The totals of each 2-letter combination were summed across all 61 variables and 30 medical records for 1830 points of comparison. These results were analyzed in R statistical analysis software (version 3.6.0; R Foundation for Statistical Computing) by creating a 3×3 table of the points of comparison and applying the *Kappa.test* package. This yielded values for relative observed agreement (*P*o), probability of chance agreement (*P*e), Cohen κ, and *P* value.

Data recorded in the review variables in the medical records were grouped together by similar features. Descriptive statistics were calculated for all available features using R, including means and ranges for numerical data and frequencies and percentages for categorical data. To anticipate, we separate results into 4 parts, including demographic characteristics, features from SDE documentation encounters, EHR features, and additional information for revewing medical records. Because this is the first summary of MMJ SDE use in this population, we also include a breakdown of patient characteristics separately based on sex as documented in the EHR.

### Ethical Considerations

Approval for this study was obtained from the Geisinger Human Research Protection Program Internal Review Board (study 2022‐0498) as part of a larger retrospective study on marijuana or cannabis documentation practices observed in the EHR. A waiver of HIPAA (Health Insurance Portability and Accountability Act) authorization and a waiver of consent were obtained to access and record patient-specific information as part of the review protocol for medical records. To protect privacy and confidentiality, access to clinical records was limited to the designated reviewers who had completed Collaborative Institutional Training Initiative training and internal compliance courses and signed acknowledgments to access protected health information. All study data were stored electronically in password-protected locations and only made available to approved study staff. No personal identifiers have been included when presenting results or submitting information for publication. No compensation was offered for participation in this study. The records generated in the completion of the review of medical records will be retained indefinitely for analysis and potential use in future studies as the protocols allow.

## Results

### Demographic Characteristics

The cohort of 156 patient records containing the table-formatted MMJ SDE consisted of more female (n=86, 55.1%) than male (n=70, 44.9%) patients and were on average 46.1 (SD 15.2) years of age with an SDE date between January 25, 2019, and May 26, 2022. The average length of their EHR was 16.0 (SD 7.8) years (Table 1). Patients were predominantly White and non-Hispanic, consistent with the demographics of this region of Pennsylvania [30]. The demographic characteristics of the cohort of medical record review were similar to the larger parent SDE population and the general Geisinger patient population (Table S1 in Multimedia Appendix 1).

**Table 1.** Demographic characteristics of a subset of patients with a table-formatted smart data element for medical marijuana in the cohort for medical record review (N=156).

		Values
**Age (years)**		
	Mean (SD)	46.1 (15.2)
	Range	20-83
**EHR**^a^ **length (years)**		
	Mean (SD)	16.0 (7.8)
	Range	1.7-33.3
**Sex, n (%)**		
	Male	70 (44.9)
	Female	86 (55.1)
**Race, n (%)**		
	Black or African American	7 (4.5)
	White	145 (92.9)
	Undisclosed or unspecified	4 (2.6)
**Ethnicity, n (%)**		
	Hispanic	6 (3.9)
	Non-Hispanic	149 (95.5)
	Undisclosed or unspecified	1 (0.6)

a EHR: electronic health record.

### Interrater Reliability and Completion Characteristics of Medical Record Reviews

The average completion time for the review protocol was 17.7 (SD 12.4) minutes per medical record (n=30; the first 15 medical records from each reviewer), with a minimum review time of 6 minutes and a maximum of 75 minutes. Standard interrater reliability calculations yielded a mean reliability percentage of 98% (SD 1.7%) when all 61 variables were compared between the initial reviewer and the third reviewer for each of the 30 comparison medical records. When only the 13 independent variables were assessed, there was a mean reliability of 95.9% (SD 4.8%). An assessment of those same 30 comparison medical records yielded a κ value of 0.97, with an odds ratio of 0.97, 95% CI of 0.95-0.98, and a *P*<.001. The κ value range is graded on a scale from 0=no agreement to 1=perfect agreement. A score of 0.81-0.99 indicates near-perfect agreement [29]. The relative observed agreement (*P*o) was .98 with a probability of chance agreement (*P*e) of .52 across 1830 points of comparison.

### Documentation Characteristics

We found that the SDE variables were coded in 2 primary locations: on the active problem list (n=17, 10.9%) and in encounter or provider notes (n=139, 89.1%; Table 2). SDE entry was largely completed by nurses and medical assistants (n=138, 88.5%) and typically within primary care settings (n=107, 68.6%). Licensed practical nurses were the provider type most frequently documenting SDEs (n=82, 52.6%), followed by medical assistants (n=33, 21.2%), registered nurses or nurse practitioners (n=23, 14.7%), and doctors or physician assistants (n=14, 9%).

When clinicians enter the SDE into the record, they also have the option to submit without entering text into all the available fields. To determine the completion of the SDE, we characterized whether each of the SDE fields contained information. Across all SDE fields, documentation was completed 76.6% (SD 23.7) of the time, with certifying provider name and location specified 61.5% (n=96) of the time, certifying conditions listed 93.6% (n=146), dispensary listed 87.8% (n=137), product type listed 92.9% (n=145), dose of product specified 30.8% (n=48), and active ingredient specified in 83.3% (n=130) of patients. Product type was variable with vape or vaporization identified in 45.5% (n=71) of documentation, dry leaf or flower in 38.5% (n=60), and tincture or drops or oil in 26.3% (n=41). A given patient can use several different products, and this variability was captured in the SDE documentation, with an average of 2 product types per patient (ranging from 1 to 6). The active ingredient was also documented, with tetrahydrocannabinol or cannabidiol in combination in the majority (n=80, 51.3%) of products, tetrahydrocannabinol only in 29.5% (n=46), and cannabidiol only in 2.6% (n=4). The dose reported was highly variable in terms of the form of measurement reported, with some specifying the amount per day, others specifying the time to use the product (ie, vape as needed, but tincture before bed), and others listed as pro re nata (meaning “as necessary”).

**Table 2.** Medical marijuana (MMJ) table-formatted smart data element (SDE) documentation completion rates and most frequent attributes, overall and by sex within the cohort for medical record review.

	All MMJ SDE charts (N=156)	Female (n=86)	Male (n=70)
SDE completion^a^ (%), mean (SD)	76.6 (23.7)	79.8 (19.0)	72.6 (28.2)
**SDE variable**			
**Location, n (%)**			
Problem list	17 (10.9)	9 (10.5)	8 (11.4)
Encounter notes	139 (89.1)	77 (89.5)	62 (88.6)
**Documenting credentials, n (%)**			
Licensed practical nurse	82 (52.6)	44 (51.2)	38 (54.3)
Medical assistant	33 (21.2)	24 (27.9)	9 (12.9)
Registered nurse or CRNP^b^	23 (14.7)	13 (15.1)	10 (14.3)
**Documenting department, n (%)**			
Family practice or primary care	107 (68.6)	62 (72.1)	45 (64.3)
Gastroenterology	17 (10.9)	8 (9.3)	9 (12.9)
Surgery	12 (7.7)	6 (7)	6 (8.6)
**Certifying providers or location**			
Included, n (%)	96 (61.5)	54 (62.8)	42 (60)
Unique, n/N (%)	66/96 (68.8)	38/54 (70.4)	36/42 (85.7)
**Dispensaries**			
Included, n (%)	137 (87.8)	81 (94.2)	56 (80)
Unique, n/N (%)	43/137 (31.4)	30/81 (37)	24/56 (42.9)
**Certifying conditions**^**c**^**, n (%)**			
Severe chronic or intractable pain	60 (38.5)	36 (41.9)	24 (34.3)
Anxiety	58 (37.2)	33 (38.4)	25 (35.7)
Posttraumatic stress disorder	27 (17.3)	17 (19.8)	10 (14.3)
**Product type**^**c**^**, n (%)**			
Vaporization	71 (45.5)	39 (45.3)	32 (45.7)
Dry leaf or flower	60 (38.5)	34 (39.5)	26 (37.1)
Tincture or drops or oil	41 (26.3)	30 (34.9)	11 (15.7)
**Dose, n (%)**			
Specified	48 (30.8)	29 (33.7)	19 (27.1)
Unspecified	108 (69.2)	57 (66.3)	51 (72.9)
**Active ingredient, n (%)**			
THC^d^ or CBD^e^	80 (51.3)	48 (55.8)	32 (45.7)
THC	46 (29.5)	24 (27.9)	22 (31.4)
CBD	4 (2.6)	4 (4.7)	0 (0)

a Individual SDE component completion that is not listed above includes certifying condition (n=146, 93.6%), product type (n=145, 92.9%), and active ingredient (n=130, 83.3%).

b CRNP: certified registered nurse practitioner.

c It is possible for patients to report more than 1 certifying condition or multiple dosage products.

d THC: tetrahydrocannabinol.

e CBD: cannabidiol.

A physician can list more than 1 certifying condition as part of their MMJ registration, which would then appear on the patient’s card. Of the 156 medical records that contained the table-formatted MMJ SDE, 224 total conditions were included, with the number of conditions ranging from 1 to 5 per person. “Severe chronic or intractable pain” was the most common certified condition, with 38.5% (n=60) of patients having this condition listed, followed by anxiety (n=58, 37.2%) and posttraumatic stress disorder (n=27, 17.3%) diagnoses. Most patients had 1 of the 24 qualifying conditions, but many patients also had a condition listed that was not one of the qualifying conditions (Table S2 in Multimedia Appendix 1).

### Summary of Features of the Encounter on the Date of the SDE

One of the anticipated features of the SDE for MMJ is that when paired with a problem list diagnosis, the diagnosis and SDE would then remain on the active problem list, enabling any clinician providing care within all departments to be immediately aware of the MMJ treatment. However, in practice, the SDE can be added anywhere in the medical record that allows free text entry and thus is not limited to the problem list diagnosis. We explored documentation and diagnoses present in the record before, during, and after the date the SDE was entered (Table 3).

We found that nearly half of the records (n=75, 48.1%) had some sort of marijuana diagnosis listed on their problem list *prior* to the SDE encounter; this diagnosis sometimes preceded the implementation of the hospital-wide SDE or could have been entered by a different clinician or at a different clinic. At the SDE encounter, MMJ or other marijuana use was never a primary reason for the visit and was the secondary diagnosis in only 17.9% (n=28) of the cohort, although some listed other substance use or abuse-related conditions, including opioid use disorder, or alcohol-induced cirrhosis or pancreatitis a primary diagnosis. We found that the marijuana use diagnosis remained on the problem list in 74.4% (n=116) of the next completed encounters.

**Table 3.** Most frequent department and diagnostic encounter characteristics surrounding table-formatted medical marijuana (MMJ) smart data element (SDE) documentation, overall and by sex.

Encounter variable	All MMJ SDE charts (N=156), n (%)	Female (n=86), n (%)	Male (n=70), n (%)
**Department**			
Family practice or primary care	107 (68.6)	62 (72.1)	45 (64.3)
Gastroenterology	17 (10.9)	8 (9.3)	9 (12.9)
Surgery	12 (7.7)	6 (7)	6 (8.6)
**Primary diagnosis** ^**a**^			
Z00-Z99	34 (21.8)	16 (18.6)	18 (25.7)
G00-G99 and M00-M99	21 (13.5)	13 (15.1)	8 (11.4)
R00-R99	21 (13.5)	15 (17.4)	6 (8.6)
K00-K95	17 (10.9)	8 (9.3)	9 (12.9)
**Secondary diagnosis** ^**b**^			
Z00-Z99	73 (46.8)	41 (47.7)	32 (45.7)
F00-F99	60 (38.5)	35 (40.7)	25 (35.7)
E00-E89	46 (29.5)	28 (32.6)	18 (25.7)
R00-R99	46 (29.5)	27 (31.4)	19 (27.1)
**Marijuana diagnosis**			
On the problem list prior to the SDE encounter	75 (48.1)	37 (43)	38 (54.3)
MMJ in secondary diagnoses of the SDE encounter	28 (17.9)	10 (11.6)	18 (25.7)
On the problem list at the next completed encounter	116 (74.4)	61 (70.9)	55 (78.6)
**“Drug use” documentation**			
Yes	66 (42.3)	40 (46.5)	26 (37.1)
Not currently	8 (5.1)	4 (4.7)	4 (5.7)
No or never	53 (34)	26 (30.2)	27 (38.6)
Marijuana specified	68 (43.6)	41 (47.7)	27 (38.6)

a E00-E89: endocrine, nutritional, and metabolic diseases; F00-F99: mental, behavioral, and other substance use disorders; G00-G99 and M00-M99: musculoskeletal and nervous system disorders; K00-K95: diseases of the digestive system; R00-R99: symptoms, signs, and abnormal findings, not elsewhere classified; and Z00-Z99: primary care, routine physical examination, or conditions not otherwise noted.

b Average number of secondary diagnoses per patient when present is 5.0 (SD 3.9; range 1-18). In total, 30 of 156 records did not have a secondary diagnosis.

Most of the primary and secondary diagnoses for the encounter where the SDEs were entered were part of routine care (physical examination, follow-up for history of a given disorder, and screening or laboratory testing), but some of these encounters included primary diagnoses for pain, diabetes, obesity, digestive, or cholesterol disorders. Common secondary diagnoses also included psychiatric disorders, endocrine system, and other signs and symptoms not otherwise classified. Overall, this indicates that MMJ use can be brought up in a variety of contexts of a primary care visit.

The social history tab of a patient’s medical record is supposed to be reviewed or confirmed at every patient visit. Questions include “Do you drink? Do you smoke? Do you have any history of drug use?”’ There is also some additional branching logic that can be used to document more specific information in free text. Of the patient cohort for medical record review, 42.3% (n=66) of the patients were marked “yes” for drug use at the SDE encounter, while 39.1% (n=61) were marked no or never or not currently. Marijuana use, specifically, was noted in the social history of 43.6% (n=68) of all records in the cohort, marked with either “yes” or “not currently.” The information noted in the social history tab of these patients suggests that providers have mixed perspectives on whether MMJ should be characterized as “drug use” in patient social history.

### Summary of Additional Features in the Context of the SDE (Before and After SDE Documentation)

We explored each patient’s health record for additional context surrounding the SDE documentation, including the first mention of MMJ use or requests for information in the record (Table 4). The first record of interest in MMJ and MMJ use was most commonly documented in primary care settings (first interest: n=77, 49.4% and first use: n=72, 46.2%), followed by surgery (first interest: n=12, 7.7% and first use: n=11, 7.1%). The first record of MMJ interest or use was mostly documented by physicians (n=61, 39.1% in each case) and physician assistants (n=32, 20.5% and n=26, 16.7%, respectively), whereas actual documentation of SDE components with more specific use information was primarily done by clinical rooming staff. We also assessed whether urine toxicology screens had been completed on the patient at prior visits and whether these were positive for marijuana. We found that 54.5% (n=85) of the cohort for medical record review had a drug screening within their health record, with 82% (70/85) of those toxicology screens being positive for marijuana. Urine drug screens could have occurred at any point in time in the patient record. The presence of urine toxicology screens in most patients in this randomly selected portion of the SDE cohort suggests that many patients were using marijuana prior to reporting the use to their doctor. These results also suggest that the EHR may be a useful source for future retrospective analysis of marijuana use in patients prior to legalization in Pennsylvania.

**Table 4.** Frequency and percentage of various problem list and nondiscrete field characteristics of patients with smart data element (SDE) documentation of medical marijuana (MMJ), overall and by sex.

	All MMJ SDE charts (N=156), n (%)	Female (n=86), n (%)	Male (n=70), n (%)
MMJ card scanned	11 (7.1)	7 (8.1)	4 (5.7)
Effect or side effect specified	62 (39.7)	34 (39.5)	28 (40)
**Any MJ**^a^ **diagnosis on the current active problem list**	127 (81.4)	68 (79.1)	59 (84.3)
MMJ	124 (97.6)^b^	67 (98.5)^b^	57 (96.6)^b^
Other MJ	3 (2.4)^b^	1 (1.5)^b^	2 (3.4)^b^
Dx date different from SDE date	88 (69.3)^b^	47 (69.1)^b^	41 (69.5)^b^
**Diagnosing department**			
Family practice or primary care	81 (63.8)^b^	44 (64.7)^b^	37 (62.7)^b^
Emergency medicine	7 (5.5)^b^	5 (7.4)^b^	2 (3.4)^b^
Surgery	3 (2.4)^b^	2 (2.9)^b^	1 (1.7)^b^
**Toxicology screens**			
Toxicology screens present	85 (54.5)	36 (41.9)	49 (70)
Toxicology screens + for MJ	70 (82.4)^c^	26 (72.2)^c^	44 (89.8)^c^
**First mention of interest in MMJ^d^**			
**Department**			
Family practice or primary care	77 (49.4)	44 (51.2)	33 (47.1)
Surgery	12 (7.7)	5 (5.8)	7 (10)
Emergency medicine	7 (4.5)	4 (4.7)	3 (4.3)
General internal medicine	7 (4.5)	5 (5.8)	2 (2.9)
**Provider type**			
Physician (MD/DO)	61 (39.1)	32 (37.2)	29 (41.4)
Physician’s assistant (PA-C)	32 (20.5)	18 (20.9)	14 (20)
Licensed practical nurse	24 (15.4)	14 (16.3)	10 (14.3)
**First record of MMJ use**			
**Department**			
Family practice or primary care	72 (46.2)	41 (47.7)	31 (44.3)
Surgery	11 (7.1)	5 (5.8)	6 (8.6)
Emergency medicine	6 (3.8)	4 (4.7)	2 (2.9)
**Provider type**			
Physician (MD/DO)	61 (39.1)	33 (38.4)	28 (40)
Physician’s assistant (PA-C)	26 (16.7)	15 (17.4)	12 (17.1)
Licensed practical nurse	24 (15.4)	14 (16.3)	9 (12.9)

a MJ: marijuana.

b Percent is out of those with a marijuana diagnosis present on the current active problem list.

c Percent is out of those with toxicology screens present.

d MMJ: medical marijuana.

## Discussion

### Principal Findings

This research had several primary goals, including to determine (1) whether the SDE was consistently capturing MMJ-related information, (2) where this SDE was being used within the medical record, and (3) whether the SDE was serving its intended purpose of making the patient’s use of MMJ easily accessible and available to clinicians across our integrated care system. We do find that customized SDEs can be used for documentation of MMJ use and that there was reasonably consistent capture of the individual SDE fields when in use. Overall, our results confirm that SDEs have the potential to make specific information relatively easy to record and find for future reference. While there was variance in use across clinicians, a concerted effort to educate clinicians and rooming staff on best practices for using the SDE may address this heterogeneity [31]. We found that the SDE was primarily used within an encounter note rather than as part of a problem list diagnosis. While the presence of the SDE within an encounter note can be helpful for research such as this, the SDE may be more useful clinically if implemented consistently as part of a problem list, so that all clinical providers can access the information quickly as part of a new encounter.

To answer our driving questions, we developed and implemented a systematic protocol for reviewing medical records. Review of medical records can be used for many purposes, including describing symptoms and prevalence of specific conditions [32,33], risk assessment [34], prediction modeling [35], and as the basis for informing natural language processing and machine learning algorithms [36,37]. We and others have started to make use of more systematic data extraction from patients’ medical records [38].

### Strengths and Limitations

We have demonstrated here and elsewhere [22,23] that establishing a review protocol for medical records can result in a highly reliable process that allows for human contextualization of information in medical records and is also scalable, with an average review of less than 20 minutes per patient. By creating the protocol with the input and guidance from clinical stakeholders, we can translate important clinical details into a format that allows for nonexperts to reliably perform the review. This point may be important for future use of this type of review protocol for medical records, particularly for reviews of medical records surrounding clinical case or control and natural language processing algorithm development [39,40]. That is, many reviews of medical records are completed to inform algorithms that are designed to automatically characterize a given patient as a case or control for a specific diagnosis (eg, schizophrenia) [41]. Validation of these algorithms typically involves confirmation as case or control by highly trained clinicians who review the entire patient’s medical record [42]. Typically, additional supporting information is not recorded as part of these reviews, and most of the details and clinical expertise required for contextualization are not documented. We show that individuals without clinical expertise can be trained in such a way to search for and identify information within the medical record that is relevant to clinical characterization. Future reviews of medical records that are focusing on diagnostic algorithm evaluation may want to use a similar process. Beyond the use for research algorithms, this procedure may be helpful for training individuals responsible for clinical documentation, such as medical scribes, as previous work indicates wide variability in scribe note structure [43].

This work is not without limitations. Reviews of medical records can be time-consuming compared to more automated electronic data extraction. While our review protocol for medical records attempts to implement a highly reliable process that is also efficient, some of the reviews of medical records can still take over an hour for a given patient. In addition, this data extraction and review focused on individuals greater than or equal to 18 years of age and thus does not include documentation of pediatric patients; future work should examine whether the SDE is used within pediatric settings with similar fidelity. Further, we have performed this review of medical records on a unique population within central and northeast Pennsylvania that has sought care at an integrated health system, Geisinger. This type of SDE documentation may not be useful if used in EHRs that are not part of integrated care settings. While this specific SDE can be incorporated into other networks that use Epic, specifically, it could be adapted into a macro or template as other EHR systems allow. Finally, the data reported here are input into the record by clinical providers and hospital staff and reported by the patient, both of which are prone to human error. The use of an SDE can guide more consistent data entry than free text alone, but we are unable to validate whether the human-entered text into the SDE is accurate, as entered. For example, some patients indicated that they were using MMJ for a condition that is not approved for MMJ treatment in Pennsylvania (eg, insomnia and depression; Table S2 in Multimedia Appendix 1). There is not currently a process in place for a clinician to verify MMJ certification using the state databases, including conditions for which a card was obtained, unless that clinician is registered with the state to endorse MMJ cards themselves. Future policy may want to consider adding this option for nonregistered clinicians if it would be useful for patient care.

### Conclusions

The model of reviewing medical records described here yielded high-quality data extraction, demonstrating its potential as a prototype for other review protocols for medical records. We find that the completeness of SDE fields was relatively high and primarily completed by clinical rooming staff. This finding suggests that improving the adoption and fidelity of SDE data collection could provide a valuable data source for consistent documentation of an alternative treatment that is typically not tracked using a formalized drug monitoring system. By leveraging this model of SDE documentation, insights can then be gained into the patterns, trends, and outcomes associated with MMJ use in a clinical setting. This information can also inform the further development of evidence-based guidelines for MMJ use and contribute to a better understanding of its therapeutic potential [44]. Additionally, the model can be adapted to study other areas of health care, facilitating the extraction of high-quality data from EHRs and contributing to advancements in clinical research more generally.

## Supplementary material

10.2196/65957 Multimedia Appendix 1 Supplemental material containing a list of all variables for reviewing medical records (Textbox S1), parent cohort and Geisinger population demographics (Table S1), and self-reported qualifying conditions (Table S2).

## Abbreviations

EHR: electronic health record
HIPAA: Health Insurance Portability and Accountability Act
*ICD*: *International Classification of Diseases*
MMJ: medical marijuana
SDE: smart data element
