# Supplementary material for: Medical Marijuana Documentation Practices in Patient Electronic Health Records: Retrospective Observational Study Using Smart Data Elements and a Review of Medical Records
Source: JMIR Form Res. 2024 Dec 23;8:e65957. doi: 10.2196/65957 (PMC11684775; doi:10.2196/65957)
Supplement: Multimedia Appendix 1 [file formative-v8-e65957-s001.docx]

**Textbox S1.** Complete list of chart review variables and branching logic.

Independent variables (n=13) listed as the main section headers, with all 61 discrete variables denoted in order by parentheses.

| 1. Any marijuana diagnosis present on the current problem list at the time of chart review? Yes/No **(1)**    1. Marijuana diagnosis name and ICD code **(2)**    2. Date the marijuana diagnosis was added to the problem list **(3)**    3. Does the diagnosis date match the SDE Date? Yes/No **(4)**       1. Marijuana diagnosis encounter          1. Department **(5)**          2. Type of clinic **(6)**          3. Provider **(7)**          4. Primary diagnosis for the SDE visit **(8)**          5. Additional diagnoses from the SDE visit **(9)**          6. “Drug Use” text **(10)**          7. “Drug Use” comment text **(11)** 2. MMJ SDE found on the problem list? Yes/No **(12)**    1. Yes       1. SDE text          1. Provider who added the MMJ SDE **(13)**          2. Certifying provider **(14)**          3. Authorized dispensary **(15)**          4. Certifying condition(s) **(16)**          5. “Dosage Product Provided” text **(17)**          6. Dose **(18)**          7. Active ingredient **(19)**       2. SDE encounter          1. Department **(20)**          2. Type of clinic **(21)**          3. Provider **(22)**          4. Primary diagnosis for the SDE visit **(23)**          5. Additional diagnoses from the SDE visit **(24)**          6. “Drug Use” text **(25)**          7. “Drug Use” comment text **(26)**    2. No       1. SDE encounter          1. Department **(27)**          2. Type of clinic **(28)**          3. Provider **(29)**          4. MMJ SDE found in an encounter note? Yes/No **(30)**             1. SDE text   Name of note author **(31)**  Role of documenting staff **(32)**  Certifying provider **(33)**  Authorized dispensary **(34)**  Certifying condition(s) **(35)**  “Dosage Product Provided” text **(36)**  Dose **(37)**  Active ingredient **(38)**   - - - 1. Primary diagnosis for the SDE visit **(39)**       2. Additional diagnoses from the SDE visit **(40)**       3. “Drug Use” text **(41)**       4. “Drug Use” comment text **(42)**  1. Was a copy of the MMJ certification card entered into the record? Yes/No **(43)**    1. Where was the MMJ card documented? **(44)**    2. Date the MMJ card was entered into the record **(45)** 2. Date of next completed encounter **(46)** 3. Marijuana diagnosis present on the problem list in the encounter note of the next visit? Yes/No **(47)**    1. Marijuana diagnosis name and ICD code **(48)** 4. Last completed encounter date **(49)** 5. First completed encounter date **(50)** 6. Number of completed toxicology screens in the patient’s lab reports **(51)** 7. Any toxicology screens positive for marijuana/cannabinoids? Yes/No **(52)**    1. Number of positive toxicology screens for marijuana/cannabinoids **(53)** 8. Any marijuana use noted? Yes/No **(54)** 9. First noted mention of MMJ interest date **(55)**    1. Department that first noted MMJ interest **(56)**    2. Provider/note author who first noted MMJ interest **(57)** 10. First noted mention of MMJ use **(58)**     1. Department that first noted MMJ use **(59)**     2. Provider/note author who first noted MMJ use **(60)** 11. Any side effects from MMJ? **(61)** |
| --- |

**Table S1.** Demographic characteristics of the parent smart data element (SDE) cohort and all eligible Geisinger patients with at least one encounter (inpatient, outpatient, emergency department, etc) at a Geisinger facility between 1/1/2013 and 6/30/2022.

|  | **Parent SDE Cohort**  **(n=2133)** | | |  | **All Eligible Geisinger**  **(n=1,762,548)** | | |
| --- | --- | --- | --- | --- | --- | --- | --- |
|  |  | **Range** | |  |  | **Range** | |
|  | **x̅ (± SD)** | **Min** | **Max** |  | **x̅ (± SD)** | **Min** | **Max** |
| **Age (years)** | 44.5 (±15) | 18 | 89+ |  | 38.0 (±25) | 0 | 89+ |
| **EHR Length (years)**^a^ | ^-^ | ^-^ | ^-^ |  | ^-^ | ^-^ | ^-^ |
|  | **n (%)** |  |  |  | **n (%)** |  |  |
| **Sex,** |  |  |  |  |  |  |  |
| Male | 953 (44.7%) |  |  |  | 840,193 (47.7%) |  |  |
| Female | 1180 (55.3%) |  |  |  | 922,199 (52.3%) |  |  |
| **Race, n (%)** |  |  |  |  |  |  |  |
| American Indian or Alaska Native | 11 (0.5%) |  |  |  | ^b^ |  |  |
| Asian | 6 (0.3%) |  |  |  | 25,961 (1.5%) |  |  |
| Black or African American | 110 (5.2%) |  |  |  | 100,816 (5.7%) |  |  |
| Native Hawaiian/ Other Pacific Islander | 6 (0.3%) |  |  |  | ^b^ |  |  |
| White | 1972 (92.5%) |  |  |  | 1,587,810 (90.1%) |  |  |
| Undisclosed or unspecified | 28 (1.3%) |  |  |  | 47,961 (2.7%) |  |  |
| **Ethnicity, n (%)** |  |  |  |  |  |  |  |
| Hispanic | 89 (4.2%) |  |  |  | 112,801 (6.4%) |  |  |
| Not Hispanic | 2018 (94.6%) |  |  |  | 1,617,725 (91.8%) |  |  |
| Undisclosed or unspecified | 26 (1.2%) |  |  |  | 32,022 (1.8%) |  |  |

^a^Not available, as the data pulled for these cohorts were limited to encounters from 2013-2022

^b^Included in the “Undisclosed or unspecified” summary

**Table S2.** Certifying conditions summary from the table-formatted smart data element (SDE) of the chart review cohort (n=156). Note: Patients can report more than one certifying condition. *A.* Pennsylvania (PA) eligible qualifying conditions for medical marijuana and count of patients with that qualifying condition self-reported in the designated smart data element field *B.* Other patient-specified conditions that are not on the list of qualifying conditions.

| **CERTIFYING CONDITIONS** |  |
| --- | --- |
| **(A) PA ELIGIBLE QUALIFYING CONDITIONS** | **N** |
| Amyotrophic lateral sclerosis | 0 |
| Anxiety disorders | 58 |
| Autism | 1 |
| Cancer, including remission therapy | 2 |
| Chronic Hepatitis C | 0 |
| Crohn’s disease | 2 |
| Damage to the nervous tissue of the central nervous system | 3 |
| Dyskinetic and spastic movement disorders | 3 |
| Epilepsy | 0 |
| Glaucoma | 1 |
| HIV / AIDS | 0 |
| Huntington’s disease | 0 |
| Inflammatory bowel disease | 1 |
| Intractable seizures | 0 |
| Multiple sclerosis | 1 |
| Neurodegenerative diseases | 0 |
| Neuropathies | 10 |
| Opioid Use Disorder | 8 |
| Parkinson’s disease | 2 |
| Post-traumatic stress disorder | 27 |
| Severe chronic pain | 59 |
| Sickle cell anemia | 0 |
| Terminal illness | 0 |
| Tourette syndrome | 0 |
| **TOTAL PA ELIGIBLE** | **178** |
|  |  |
| 1. **OTHER PATIENT-SPECIFIED CONDITIONS** | **N** |
| Arthritis | 6 |
| Bipolar Disorder | 1 |
| Borderline Personality Disorder | 1 |
| Degenerative Disc Disease | 1 |
| Depression | 12 |
| Digestive Issues | 1 |
| Fibromyalgia | 3 |
| Gastritis | 1 |
| Insomnia/Sleep Disorders | 12 |
| Lupus | 1 |
| Mental Health | 1 |
| Nausea | 3 |
| Polycystic Ovary Syndrome (PCOS) | 1 |
| Quadriplegia | 1 |
| Stress | 1 |
| **TOTAL PATIENT-SPECIFIED** | **46** |
|  |  |
| **TOTAL CONDITIONS REPORTED (TOTAL A + TOTAL B)** | **224** |
